# Dynamical properties of maps fitted to data in the noise-free limit

**DOI:** 10.1080/17513758.2013.804127

**Published:** 2013-06-14

**Authors:** Torsten Lindström

**Affiliations:** Department of Mathematics, Linnaeus University, S-35195 Växjö, Sweden

**Keywords:** deterministic chaos, model selection, regression model, time-series, threshold autoregressive model, skew tent map, uniqueness of periodic attractors, difference equation, bifurcation diagram

## Abstract

We argue that any attempt to classify dynamical properties from nonlinear finite time-series data requires a mechanistic model fitting the data better than piecewise linear models according to standard model selection criteria. Such a procedure seems necessary but still not sufficient.

## 1. Introduction

May and Oster [23] pointed out the possibility that simple nonlinear ecological models may possess very complicated dynamics. However, mentioning that interesting phenomena might occur does not mean proving their existence. After May and Oster's [23] paper, there were several attempts to corroborate chaos in ecology by fitting nonlinear models to population data. One of the first studies was made by Hassell *et al.* [13] and their conclusion was that most data corresponded to parameter values outside the chaotic regime. Reasons for such observations have been discussed since then (see e.g. [3, 25, 26]). One of the most intriguing issues in this context is the methodological one: Does there exist any method allowing for some kind of classification of the long-run qualitative behaviour from the time-series data? And if one exists, what amount of data and what kind of prior information regarding the data is then needed?

Morris [24] illustrated at least three problems that might arise as attempts to derive the long-run qualitative behaviour from time-series data are analysed and the issue is still not closed (see [2, 17, 29, 35] and references therein). In this paper, we stress that models ranked by model selection criteria such as Akaike Information Criterion (AIC) or generalized cross-validation (GCV) (see e.g. [4, 9, 14, 28]) optimize various short-term predictive properties. Highly ranked models may, therefore, display long-run dynamical properties deviating from those of the time-series. Thus, additional information must be provided in order to ensure topological equivalence.

State space reconstruction theorems, cf. Takens [31] and Deyle and Sugihara [6], ensure that positive Lyapunov exponents can be computed from infinite quantities of deterministic data. A method for the classification of dynamical behaviour for time-series cannot rely on such prior information. Measurements are always finite in number and a priori information regarding the sign of the Lyapunov exponent cannot be assumed to exist if a topological classification remains an objective. We find an analysis in the noise-free limit particularly interesting in the light of the expanding body of literature concerning noise reduction in general, see e.g. Stark [30] and Han and Chang [11].

**Figure 1.**
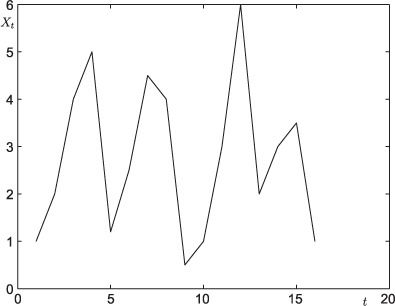
One-observable census-data for some population.

Our paper is organized as follows: We commence with some background of the problem and mechanistic modelling. We then introduce data-based modelling and the dynamics of piecewise linear maps. We define stability switches in one dimension and discuss the information available in the noise-free limit of finite one-observable time-series data. The main referenceis Lindström [20].

## 2. Nonlinearity necessary

Consider an one-observable time-series, like the one in Figure 1. The observed time-series has the following properties: It is of *finite size* and usually short. It may be oscillatory and potentially chaotic. Aswe are considering graphsofone-observable census-data, like Figure 1, questions concerning the mechanisms behind the observed behaviour may arise. We ask whether some interactions between species occur and questions regarding at what level this interaction occurs might follow. If we are observing some herbivores, we may ask whether the oscillations are due to vegetation–herbivore or herbivore–carnivore interaction, cf. Turchin *et al.* [34] and Lindström [19]. Alternative explanations like resource partitioning, or noise, may exist. In ecological terms, linear models combined with noise are not a sufficient explanation. Linear models combined with noise may lead to a negative number of individuals. Moreover, if this possibility is removed by introducing log-linear models, then the populations may start producing their own limiting resources at low densities. Absurd predictions of linear and log-linear ecological models have been mentioned before, see e.g. Hassell [12] or Lima [18].

## 3. Data-based model selection and consequences of nonlinearity

In many cases, we do not have good ideas regarding the reason behind possible population oscillations. But we still want to find out a function being able to predict the points in the data-set. It is difficult to determine a suitable class of functions to be used to explain the data. Polynomials can be used on finite intervals (cf. [7]) and they range from the response-mean models up to interpolation models. If we choose to work with response-mean models, then all oscillations are explained as noise and there are no trends. In the interpolation extreme, all oscillations are explained as trends and there is no noise. We want to select some functions between these two extremes and there exist several methods for doing this, see e.g. Hastie and Tibshirani [14] or Burnham and Anderson [4]. The objective is to find some functions separating the trends in the data from the noise.

One method for doing this is to train the function to predict the data. We delete one point in the data set and use the remaining ones to predict the deleted point. We repeat this procedure and delete one point at a time. We select the function between response mean and interpolation that is the best one to use the remaining points to predict the deleted points and the procedure is called cross-validation. There are many variants of this procedure and historically GCV was used in order to save computing efforts. Nowadays, new methods save computing time, but GCV is still used to take into account outliers in the data, cf. Green and Silverman [9].

A different method is the AIC [1]. It has a number of nice theoretical properties, see Burnham and Anderson [4] and modifications for small sample sizes exist in the linear case, cf. Hurwich and Tsai [15]. Assume a true model *f* and consider a set of candidate models *g_i_* (*x* | *θ*). The candidate models can now be ranked with respect to *f* according to


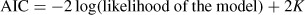


or


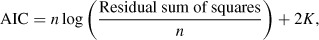


where the last formula applies to the least-squares method and assumes normally distributed errors. The number *K* is the total number of estimated parameters including the intercept and the variance. AIC has the property that it will always rank the true reality model as the best model if it is within the model set. Such assumptions cannot be made in general. The major drawback of all these methods is that second derivatives are penalized in the procedure. Thus, if the true reality is nonlinear, the true reality is penalized too (cf. [14]). We concluded in Section 1 that neither linear nor log-linear models are realistic alternatives in ecology.

## 4. One-dimensional stability switches

We introduce the Sheperd [27]


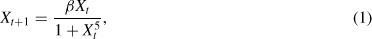


model as a prototype model. If *X_t_* ≥ 0, then Equation (1) is unimodal and it has negative Schwatzian derivative (cf. [10]). Thus, it has a unique periodic attractor (if one exists) and undergoes the period doubling route to chaos cf. Devaney [5]. On log-scale (1) takes the form


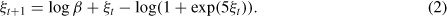


**Figure 2.**
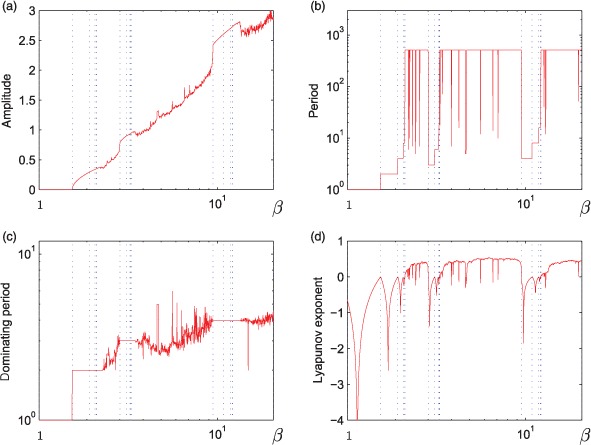
Dynamical properties of the model (2): (a) mean amplitude, (b) periods if any below 512, (c) dominating period, and (d) Lyapunov exponent. Dotted lines indicate the main bifurcations.

Work on the log-scale has several advantages. First, if we add noise, then the addition of noise cannot cause negative numbers of individuals in the data. Second, if we fit models to data generated by Equation (2), then the fitted models cannot predict negative numbers of individuals, either. Third, we note that Equation (2) is concave down. Thus, if piecewise linear models are fitted to the data in the noise-free limit, then these piecewise linear models are continuous. Model (2) has the dynamical properties depicted in Figure 2. In Figure 2(a), we can follow the amplitude of the solutions and we see the that it gets its first positive value after the first period doubling bifurcation. In Figure 2(b)–(d), we note the complete match of periodic behaviour and negative Lyapunov exponents. Figure 2(c) demonstrates the dominating period of the solution or the maximum of the Fourier transform of the solution.

## 5. Continuous TAR-models or skew tent maps

Continuous piecewise linear models are an important class of models and we are going to examine the properties of these models carefully in the single threshold case. Depending on literature, these maps have different names. In the statistical literature, the maps are usually called threshold autoregressive (TAR) models (cf. [32]) and in the dynamical systems literature they are referred to as skew tent maps, too, see Lindström and Thunberg [22] and references therein.

A continuous piecewise linear model with one threshold has four parameters and can be stated as


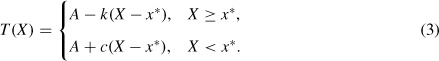


**Figure 3.**
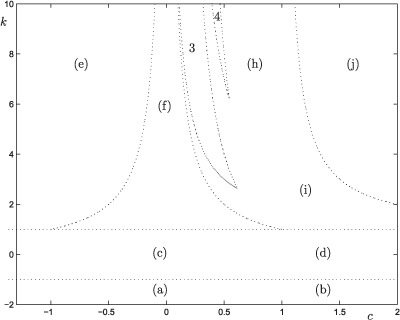
Bifurcation diagram of skew tent maps. The regions corresponding to different dynamical behaviour are plotted with dotted lines. (a) All solutions explode to infinity, (b) some solutions explode to minus infinity, the rest to infinity, (c) fixed point globally stable, (d) some solutions tend to the fixed point, the rest to minus infinity, (e) unbounded oscillations, (f) a two-periodic solution that is globally stable almost everywhere exists, corresponding regions for three-and four-periodic almost everywhere globally stable solutions are denoted with 3 and 4, respectively, (h) almost all solutions are attracted to a bounded chaotic solution, (i) almost all solutions starting within a special interval are attracted to a bounded and chaotic solution, solutions outside the interval tend to minus infinity, and (j) almost all solutions tend to minus infinity.

The parameters *A* and x* can be eliminated by linear coordinate transformations, leaving the slope parameters *c* and *k* as the essential ones (for details, see [22]). Disregarding the most simple and analogous cases, we shall focus on


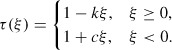


The essential dynamics of the map ξ_*t*+1_ = τ (ξ_*t*_) is that:

(1) Periodic attractors are unique, if they exist.
(2) There exist no period doubling bifurcations.
(3) Chaos bifurcate typically from an interval of neutrally stable periodic solutions.

Therefore, the map may be unimodal, but it does not have negative Schwartzian derivative, cf. Devaney [5]. It is remarkable that it is still possible to prove uniqueness of the periodic attractors. The details of the bifurcation diagram are depicted in Figure 3. Note that all curves depicted in the bifurcation diagram can be computed analytically, see Lindström and Thunberg [22].

## 6. Candidate models in the noise-free limit

We proceed by fitting continuous piecewise linear models (3) to data generated by the Sheperd model (2). Both models have unique periodic attractors. Therefore, if the itinerary ends up at a periodic attractor for the Sheperd map for a certain parameter value, no other periodic attractors can exist for the same parameter value. Therefore, possible initial value dependence is restricted. The same holds for piecewise linear maps with one threshold.

**Figure 4.**
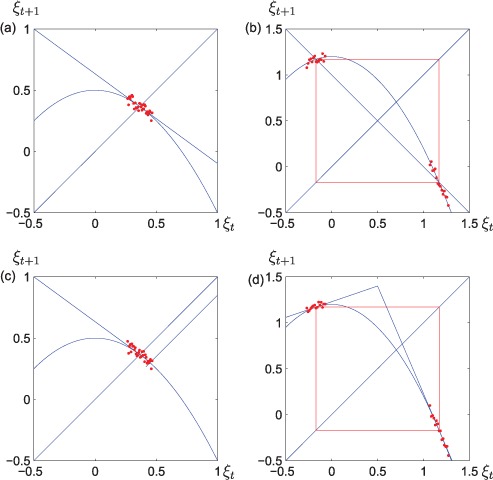
Maps fitted to a nonlinear ‘true reality’ in different cases: (a) a linear map fitted to noisy data near an attracting fixed point, (b) a linear map with neutrally stable period two solutions is fitted to data near an attracting period two orbit, (c) a piecewise linear map may over-fit the data near an attracting fixed point, and (d) a piecewise linear map is fitted to noisy data in the vicinity of an attracting two periodic solution. In the last case, the stability properties of the period two solution are preserved, sufficient amounts of data exist and the piecewise linear maps has the required flexibility.

We expect the fitting procedure to behave in the following way in the noise-free limit: If Equation (2) has an attracting fixed point for some parameter value, then the data-sets should be located close to the fixed point. If a linear map is fitted to the data-set, it is expected to be tangent to the Sheperd map. Thus, the stability properties of the fixed point of the Sheperd map are reflected by the fitted linear map, see Figure 4(a). Again, if a linear map is fitted to a period two orbit, then it tends to get the locations correctly, but the linear map has not enough flexibility to catch the stability properties of the period two orbit. Hence, a neutrally stable period two solution is fitted to an attracting period two solution and this is the first example of what we shall call a stability switch, see Figure 4(b). If a single breakpoint piecewise linear map is fitted to data in the vicinity of an attracting fixed point, then over-fitting is possible, see Figure 4(c). On the other hand, a fitted single breakpoint piecewise linear map describes both the location and the stability properties of an attracting period two solution, cf. Figure 4(d). If we have a concave nonlinear true reality and a model set consisting of linear maps and continuous piecewise linear maps with one breakpoint, then we hope that model selection criteria such as AIC should be able to select the linear map in the fixed point case (Figure 4(a)) and the single breakpoint continuous piecewise linear map in the period two case (Figure 4(d)). Indeed, the results in [20] show this to be the case.

**Figure 5.**
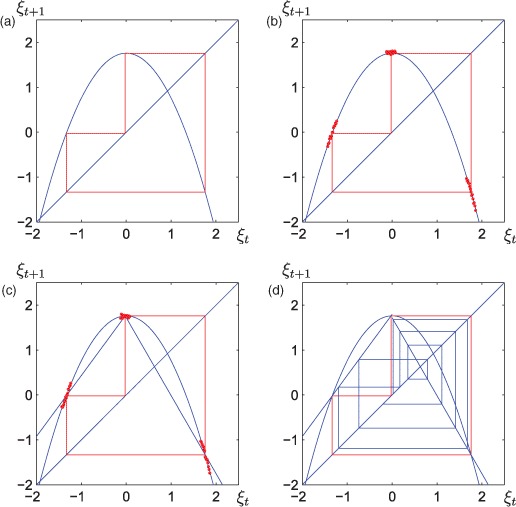
Special study of a period three case: (a) cobweb-diagram demonstrating an attracting period three-cycle of the true reality map, (b) a small amount of noise is added and the data are located in the vicinity of the period three orbit, (c) a continuous piecewise linear map is fitted to the data in the noise-free limit, and (d) the solutions are diverging from the unstable period-three orbit of the piecewise-linear map.

The results are even more intriguing in the higher periodic cases, see Figure 5, where we start from an attracting period three orbit of the true reality map in Figure 5(a). A small amount of noise is added to simulate the noise-free limit in Figure 5(b). A continuous piecewise linear map is fitted to the data but now its flexibility does not allow the slopes of the true reality map to be approximated correctly, see Figure 5(c). The location of this orbit is, however, approximated correctly. Therefore, the continuous piecewise linear map has a period three orbit, but this orbit may be unstable, as it is the case in Figure 5(d). The rich support of unstable periodic orbits in potentially chaotic nonlinear maps (cf. [5]) is therefore used when using nonlinear maps for modelling purposes. This does not mean that the long-run dynamics is modelled correctly.

It seems that already Turchin and Taylor [33] noted stability switchesin this context, the question is whether they treated them adequately. In fact, they note trajectories escaping to infinity. Our interpretation of their observation is that the best short-term predictor of the attractor data is a repelling invariant set in the basin of attraction of infinity. In cases that possess several attractors, the basin of attraction boundaries may have a complicated structure, cf. Edmunds [8]. Determining in what basin a repelling set is located after a stability switch is therefore not a trivial problem.

Our results in [20] show that data-based modelling starts behaving exactly in the above-mentioned way when the dynamical properties of the true reality map begin exceeding the flexibility of the fitted piecewise linear map. Moreover, false nonlinear maps do not trace the dynamics of the true reality model better, see e.g. Kendall [16] and our analysis of the Ricker map in [20].

## 7. Discussion

A natural question is now: Can we repair the above-mentioned stability switches by fitting more complex piecewise linear maps to the data? The answer is that these stability switches will occur anyway because a finite data-set was assumed and periods of arbitrary length were possible. A long period cannot be well populated by a finite data-set, and any data-based modelling method will select a model with a limited complexity. In any case piecewise linear models seem to trace the dynamics of the data-sets far better than possibly false nonlinear ones. Therefore, our study indicates that a nonlinear model is not justified unless it is ranked better than piecewise linear candidates. The study can be extended to more complex models like food-chains, too, see Lindström [21].

## References

[1] Akaike H., Petrov B.N., Csaki F. (1973). Information theory and an extension of the maximum likelihood principle. 2nd International Symposium on Information Theory.

[2] Beninka E., Huisman J., Heerkloss R., Jöhnk K.D., Branco P., Van Nes E.H., Scheffer M., Ellner S.P. (2008). Chaos in long-term experiment with a plankton community. Nature.

[3] Berryman A.A., Milstein J.A. (1989). Are ecological systems chaotic – and if not, why not?. Trends Ecol. Evol..

[4] Burnham K.P., Anderson D.R. (2002). Model Selection and Multimodel Inference.

[5] Devaney R. (2003). An Introduction to Chaotic Dynamical Systems.

[6] Deyle E.R., Sugihara G. (2011). Generalized theorems for state space reconstruction. PLoS One.

[7] Dzyadyk V.K., Shevchuk I.A. (2008). Theory of Uniform Approximation of Functions by Polynomials.

[8] Edmunds J.L. (2007). Multiple attractors in a discrete competition model. Theor. Popul. Biol..

[9] Greenand P.J., Silverman B.W. (1994). Nonparametric Regressionand Generalized Linear Models. Monographs on Statistics and Applied Probability,.

[10] Guckenheimer J., Oster G., Ipaktchi A. (1977). The dynamics of density dependent population models. J. Math. Biol..

[11] Han X., Chang X. (2013). An intelligent noise reduction method for chaotic signals based on genetic algorithms and lifting wavelet transforms. Inf. Sci..

[12] Hassell M.P. (1974). Density dependence in single-species populations. J. Animal Ecol..

[13] Hassell M.P., Lawton J.H., May R.M. (1976). Patterns of dynamical behaviour in single-species populations. J. Animal Ecol..

[14] Hastie T.J., Tibshirani R.J. (1990). Generalized Additive Models.

[15] Hurvich C.M., Tsai C.-L. (1989). Regression and time series model selection in small samples. Biometrika.

[16] Kendall B.E. (2001). Cycles, chaos, and noise in predator-prey dynamics. Chaos Solitons Fractals.

[17] Li B., Wang Y.-Z., Rong X.-X., Su J., Wang R.-K. (2010). Does chaos exist in ecology? Evidence from a rodent population. Int. J. Nonlinear Sci. Nonlinear Simul..

[18] Lima M. (2001). The dynamics of natural populations: Feedback structures in fluctuating environments. Revista chilena de historia natural.

[19] Lindström T. (2002). On the dynamics of discrete food-chains: Low- and high-frequency behavior and chaos. J. Math. Biol..

[20] Lindström T. (2009). Detecting chaos requires careful analysis of nearly periodic data. Chaos Soliton. Fract..

[21] Lindström T. (2009). Stability switches in discrete food-chain problems. Int. J. Bifurcation Chaos.

[22] Lindström T., Thunberg H. (2008). An elementary approach to dynamics and bifurcations of skew tent maps. J. Differential Equations Appl..

[23] May R.M., Oster G.F. (1976). Bifurcations and dynamic complexity in simple ecological models. Am. Naturalist.

[24] Morris W.F. (1990). Problems in detecting chaotic behavior in natural populations by fitting simple discrete models. Ecology.

[25] Nisbet R., Blythe S., Gurney B., Metz H., Stokes K. (1989). Avoiding chaos. Trends Ecol. Evol..

[26] Rai V., Upadhyay R.K. (2006). Evolving to the edge of chaos: Chance or necessity?. Chaos Soliton. Fract..

[27] Sheperd J.G. (1982). A family of general production curves for exploited populations. Math. Biosci..

[28] Shih S.H., Tsokos C.P. (2009). A new forecasting model for nonstationary environmental data. Nonlinear Anal..

[29] Singh B.K., Parham P.E., Hu C.-K. (2011). Structural perturbations from population skeletons: Transient dynamics, coexistence of attractors and the rarity of chaos. PLoS One.

[30] Stark H.-G. (2005). Wavelets and Signal Processing: An Application Based Introduction.

[31] Takens F., Rand D.A., Young L.S. (1981). Detecting strange attractors in turbulence. Lecture Notes in Mathematics.

[32] Tong H. (1990). Non-linear Time Series.

[33] Turchin P., Taylor A.D. (1992). Complex dynamics in ecological time series. Ecology.

[34] Turchin P., Oksanen L., Ekerholm P., Oksanen T., Henttonen H. (2000). Are lemmings prey or predators?. Nature.

[35] Upadhyay R.K. (2009). Observability of chaos and cycles in ecological systems: Lessons from predator-prey models. Int. J. Bifurcation Chaos.

